# Prognostic implication and immunotherapy response prediction of a novel ubiquitination-related gene signature in liver cancer

**DOI:** 10.18632/aging.205926

**Published:** 2024-06-12

**Authors:** Re-Guang Pan, Jingyao Zhou, Xiao-Wu Wang, Xi-Kai Cen, Yu-Ping Zhou, Yang-Yang Guo, Xue-Feng Feng

**Affiliations:** 1 Department of Hepatobiliary Surgery, The First Affiliated Hospital of Ningbo University, Ningbo, Zhejiang, China; 2 Department of Pharmacy, Taizhou Central Hospital, Taizhou, Zhejiang, China; 3 Department of Thyroid and Breast Surgery, The First Affiliated Hospital of Ningbo University, Ningbo, Zhejiang, China; 4 Department of Burns and Skin Repair Surgery, The Third Affiliated Hospital of Whenzhou Medical University, Ruian, Zhejiang 325200, China; 5 Department of Gastroenterology, The First Affiliated Hospital of Ningbo University, Ningbo, Zhejiang, China

**Keywords:** ubiquitination, liver cancer, gene signature, prediction, prognosis, response to immunotherapy

## Abstract

HCC, also known as hepatocellular carcinoma, is a frequently occurring form of cancer with an unfavorable prognosis. This research constructed a prognostic signature related to ubiquitination and investigated its correlation with the response to immunotherapy in HCC. The Molecular Signatures Database provided a compilation of genes associated with ubiquitination. A gene signature related to ubiquitination was obtained through Cox regression using the Least Absolute Shrinkage and Selection Operator method. The genetic factors CPY26B1, MCM10, SPINK4, and TRIM54 notably impacted the outcomes of HCC. The patients were divided into two groups: one group had a high risk of poor survival while the other had a low risk but a greater chance of controlling HCC progression. Both univariate and multivariate analyses using Cox regression found the risk score to be an independent predictor of HCC prognosis. Gene set enrichment analysis (GSEA) indicated enrichment in cell cycle and cancer-related microRNAs in high-risk groups. The tumor microenvironment (TME), response to immunotherapy, and effectiveness of chemotherapy medications positively correlated with the risk score. In the high-risk group, erlotinib showed higher IC50 values compared to the low-risk group which exhibited higher IC50 values for VX-11e, AKT inhibitor VIII, AT-7519, BMS345541, Bortezomib, CP466722, FMK, and JNK-9L. The results of RT-qPCR revealed that the expression of four UEGs was higher in tumor tissue as compared to normal tissue. Based on the genes that were expressed differently and associated with ubiquitination-related tumor categorization, we have developed a pattern of four genes and a strong nomogram that can predict the prognosis of HCC, which could be useful in identifying and managing HCC.

## INTRODUCTION

Liver cancer is the sixth most commonly diagnosed cancer and the third deadliest [1, 2]. There are approximately 906,000 new cases of liver cancer and 830,000 deaths globally every year [3]. Primary liver cancer is the fifth most common cancer globally and a leading cause of cancer-related deaths. Asian countries have the highest incidence rates [4]. Hepatocellular carcinoma (HCC) is the most common type of liver cancer, and it is responsible for the majority of liver cancer cases. As a result, the incidence and mortality rates of liver cancer are high [5, 6]. However, over the last twenty years, there has been significant progress in the management of HCC in mainland China [7, 8]. In China, a meta-analysis of 11 studies found a 5-year survival rate of 14.8% [9]. A retrospective study of 2887 HCC cases diagnosed between 2002 and 2015 indicated a median survival time of 9.0 months [10].

The process of ubiquitination involves attaching a ubiquitin molecule to the substrate and plays a crucial role in the adaptive mechanisms of highly-invasive, rapidly-multiplying cancer cells [11–13]. E3 ligase is necessary for ubiquitination, as it controls a wide range of cellular functions by transferring ubiquitin (Ub) to the substrate. The control mechanism that determines the exactness of the procedure is strongly linked to the development of cancer and the advancement of tumors [14]. Furthermore, the ubiquitination process could indicate the breakdown through proteasomes (degradative ubiquitination) or modification of function (regulatory ubiquitination) [15]. The previous studies extensively explored the incorporation of transcriptomics and ubiquitination, using statistical testing and machine learning. This led to significant advancements and established ubiquitination as a crucial characteristic of cancer [13, 16, 17]. Aberrations in the ubiquitination process may contribute to the diverse nature of lung cancer. Targeting ubiquitin could lead to innovative and effective cancer treatments [18, 19]. Therefore, it is crucial to detect resilient tumor-related ubiquitination biomarkers, which have the potential to enhance the detection and prediction of HCC and aid in the advancement of novel therapeutic approaches.

## METHODS

### Data profiles

The transcription statistics data, which is measured in fragments per kilobase of exon model per million reads mapped (FPKM), along with important clinical information such as age, sex, clinic grade, pathological stage, and T stage, were obtained from the Cancer Genome Atlas (TCGA) LIHC project. If the clinical grade cannot be determined as GX, it is highly differentiated as G1, moderately differentiated as G2, poorly differentiated as G3, and undifferentiated as G4. Pathological stage I refers to tumors that exceed 2 cm, but none of them affect blood vessels in the liver. They can spread to veins, arteries, and bile ducts. Stage II refers to tumors with a size exceeding 2 cm but not exceeding 5 cm, which have spread to blood vessels, veins, and arteries without lymph nodes or distant metastasis. Phase III tumors have a diameter greater than 5 cm and have not spread to lymph nodes, but may have spread to nearby organs or peritoneum without distant metastasis. Phase IV tumors have already shown intrahepatic metastasis or spread to surrounding organs and lymph nodes, or distant organ metastasis. You can find the relevant details in Supplementary Table 1. To validate the signature, we obtained an additional microarray dataset from the database of the International Cancer Genome Consortium (ICGC). We utilized the HCC models proposed by Liu et al., Zhang et al., Xie et al., and Li et al. to test and demonstrate the advantages of our prognostic signature related to ubiquitination. [20–23].

### Analysis of genes related to ubiquitination using consensus clustering

A set of 79 genes related to ubiquitination (URGs) was obtained from the MSigDB database. You can find these genes listed in Supplementary Table 2. To categorize HCCs based on the expression of URGs, an unsupervised clustering analysis was performed using the ConsensusClusterPlus R package. This analysis aimed to separate the HCCs into distinct clusters [24].

### The correlation among molecular subtypes, clinical characteristics, and prognosis of HCC

To determine the medical relevance of the two subcategories discovered through harmony gathering, we examined the relationships among the different molecular subtypes, clinicopathological characteristics, and prognostic outcomes. The patient’s attributes included age, sex, clinic grade, pathological stage, and T stage. To assess the differences in overall survival (OS) between various subtypes, we used Kaplan-Meier curves generated by the R packages ‘survminer’ and ‘survival’ [25].

### Clusters associated with ubiquitination in the tumor microenvironment

The presence of immune cell infiltration in the tumor microenvironment (TME) of ubiquitination-associated clusters was determined using the CIBERSORT algorithm. Next, the limma algorithm was used to identify differences in immune and stromal cell categories, which were then presented through violin plots [26]. The R software packages, limma, and CIBERSORT, were utilized to display this information.

### Creating and verifying a prognostic marker for HCC

We received raw mRNA expression data from the TCGA-LIHC project, which we then normalized. To identify the downstream genes affected by URGs, we analyzed the ubiquitination-related clusters using the R limma package. We considered statistical significance with *p*-values less than 0.05 and absolute log2 fold change greater than 1 [26]. Univariate Cox regression analyses were used to classify prognostic DEGs at *p* < 0.05. Then, LASSO regression and multiCox analysis were performed to establish a predictive signature for HCC [27].


$$\text{prognosis index (PI) = }\sum_{i=1}^{n}\text{Coe}\;f(i)\times \text{Expr}(i)$$

Patients from TCGA-LIHC were divided into low and high-risk subgroups based on their median risk score. The training set was randomly selected from half of the TCGA-LIHC dataset, while the other half of the patients were assigned as the internal test set. We acquired an extra microarray dataset (ICGC-LIRI-JP) from the ICGC database to validate the ubiquitination-related signature as the external test set. To compare the survival between two groups in the training and test sets, we utilized the Kaplan-Meier plotter from the R survival package. Receiver operating characteristic (ROC) curves were used to assess the prognostic capability of the ubiquitination-associated pattern for hepatocellular carcinoma (HCC) survival at 1-, 3-, and 5-year intervals with area under the curve (AUC) benchmarks [28]. The TCGA-LIHC dataset was used to present the survival outcome of individuals with HCC. To differentiate between low- and high-risk HCC patients, their separation was assessed using principal component analysis (PCA) and t-distributed stochastic neighbor embedding (t-SNE) analysis [29, 30]. To better understand the predictive properties of the signature, we performed univariate and multivariate Cox regression analyses to identify the independent risk factors for HCC. The variables taken into account in the analysis were the risk score, age, gender, clinical grade, pathological stage, and T stage.

### Creating and validating the nomogram

To improve the predictive significance, we constructed a nomogram using ubiquitination-associated genes and novel features. We used the R packages ‘rms’ and ‘regplot’ for this purpose [31]. To predict patient survival rates at 1, 3, and 5 years, the nomogram was utilized. The accuracy of the nomogram was assessed using calibration curves [32]. To evaluate the robustness of the predictive nomogram, we conducted ROC analyses [33].

### Identification and annotation of DEG functions

To investigate the possible cellular roles and enriched pathways of downstream genes of URGs, analyses of functional enrichment were conducted on the DEGs using the ‘cluster profiler’ R package [34], which included GO and KEGG. By utilizing GSEA, we were able to identify pathways that exhibited differential enrichment in low- and high-risk HCC.

### Analysis of subgroups with low and high risk of HCC

By examining the link between ubiquitination-related patterns and clinical characteristics, we could gain valuable insight into the risk disparities among subgroups of HCC patients. To achieve this, we thoroughly evaluated age, gender, clinic grade, pathological stage, and T stage. With this information, we could better understand the factors that contribute to risk. The Kaplan-Meier method was utilized to investigate the differences in survival rates among HCC patients with varying risk levels across different subcategories. The results provided valuable insights into the disparities in HCC patient outcomes.

### Analysis of TME and the response to immunotherapy in HCC with varying risk levels

Pearson’s coefficient was utilized to assess the relationship between the ubiquitination-associated pattern and genes involved in immune regulation, immune checkpoint, and diverse immune cell types. Timer, Xcell, Quantiseq, MCPcounter, EPIC, and Cibersort procedures [35–39] were utilized to create a bubble chart illustrating the infiltration of immune cells in low- and high-risk HCC. The calculation of TIDE scores was performed to forecast the response to immunotherapy. The effectiveness of a possible anti-PD-1 treatment was assessed by analyzing the IMvigor 210 cohort [40].

### Assessment of the susceptibility to drugs in hepatocellular carcinoma (HCC) with varying levels of risk

To forecast the sensitivity of drugs in HCC patients with varying risks, the R package called ‘pRRophetic’ was utilized [41]. The raw dataset was obtained from the GDSC database, with the half-maximal inhibitory concentration (IC50) serving as the reference point.

### RT-qPCR

TRIzol was utilized to extract Total RNA from both Tumor tissue and normal tissue, which was then reverse-transcribed into cDNA. The qPCR master mix was utilized for RT-qPCR. The experiments were repeated three times at least, and the Ct values were standardized to the internal reference gene, GAPDH, using the 2^−ΔΔCT^ technique.

The following primer sequences were used: 5′-GGAG CGAGATCCCTCCAAAAT-3′ (forward) and 5′-GGCT GTTGTCATACTTCTCATGG-3′ (reverse) for GAPDH; 5′-GGCAACGTGTTCAAGACGC-3′ (forward) and 5′- TGCTCGCCCATGAGGATCT-3′ (reverse) for CYP26B1; 5′-TGTCCCTGCGCTACCAAGA-3′ (forward) and 5′-GATGAGCTTTTGGGATCTGGAG-3′ (reverse) for MCM10; 5′-CAGTGGGTAATCGCC CTGG-3′ (forward) and 5′-CACAGATGGGCATTCT TGAGAAA-3′ (reverse) for SPINK4; and 5′-ATC GTGCAGGCATGAGGTTG-3′ (forward) and 5′-CCTC GCACATGAGGTGCTG-3′ (reverse) for TRIM54.

### Immunohistochemistry

Immunohistochemistry was utilized to investigate the expression of CYP26B1, MCM10, SPINK4, and TRIM54 in liver tissue. Tumor tissues were collected from patients after surgery confirmed HCC by pathology. Normal tissues were collected from patients after surgery confirmed not HCC by pathology. All patients were informed consent before surgery. Formalin-fixed, paraffin-embedded sections (5 μm) were treated with xylene to remove the wax and subsequently rehydrated in a descending gradient of alcohol solutions. Sections were then incubated with sodium citrate (0.01 mol/L, pH 6.0) at 100°C for 15 minutes to repair the antigen. To eliminate the influence of endogenous peroxidases, 3% hydrogen peroxide was used. The sections were then blocked and incubated with antibodies overnight at 4°C. Following this, the sections were washed and incubated with secondary antibodies for 2 hours at 37°C. The immunocomplexes were visualized as brown pigments using diaminobenzidine. After staining with hematoxylin, the slides were dehydrated with alcohol and cleared in xylene. Representative images were captured with light microscopy.

### Statistical analysis

All calculations and statistical data were generated with the R software (version 4.0.3). *p* < 0.05 was deemed to be statistically significant.

## RESULTS

### Detecting clusters associated with ubiquitination in HCC

**Figure 1:**
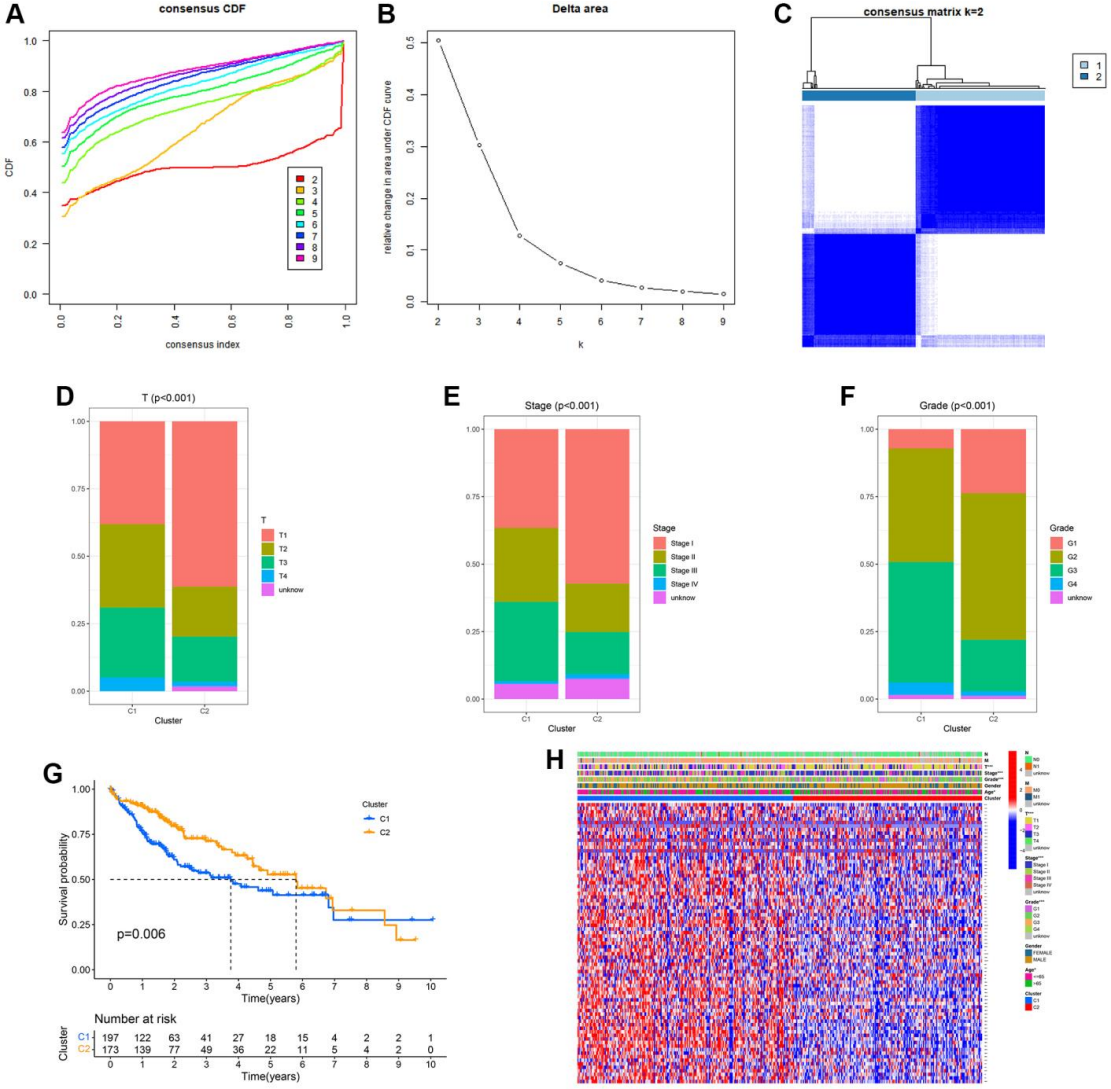
**Clinical pathology and prognostic value of two distinct subgroups of patients divided by consistent clustering.** (**A**) Consensus CDF in consistent clustering (k = 2–9). (**B**) Delta area curve of consensus clustering. (**C**) Consensus clustering matrix. (**D**–**F**) Differences in clinical pathology features between the two clusters. C1, cluster 1; C2, cluster 2. (**G**) Kaplan–Meier (K–M) survival analysis of the three subgroups. (**H**) Heatmap of DEG expression profiles in three subgroups.

To comprehend the importance of URGs in the process of tumor development, we employed a consensus clustering algorithm to categorize HCC based on the mRNA expression of 79 URGs. Figure 1A, 1B displayed the agreement cumulative distribution function (CDF) of reliable clustering for values of k ranging from 2 to 9. The cohort in our dataset was organized into clusters 1 and 2 (Figure 1C), with k = 2 being deemed the optimal selection. We separated the HCCs from the TCGA-LIHC project into two clusters. Compared with cluster 1, cluster 2 has a better prognosis. Cluster 1 showed higher clinical grade, pathological stage, and T-stage, indicating a possible association between cluster 1 and increased tumor stage in HCC. According to the data presented in Figure 1D–1F, cluster 2 exhibited a stronger association with a lower T stage, pathological stage, and clinic grade compared to cluster 1. Cluster 2 exhibited a greater survival rate than cluster 1 based on the Kaplan–Meier curve (*p* = 0.006; Figure 1G), indicating that lower T staging, pathological staging, and clinical grading are associated with good prognosis. Furthermore, the clinicopathological distributions of the various HCC subtypes demonstrated significant variations in URG expression and clinicopathological features (Figure 1H), which reminded us to further study the relationship between URG expression and the prognosis of HCCs.

**Figure 2:**
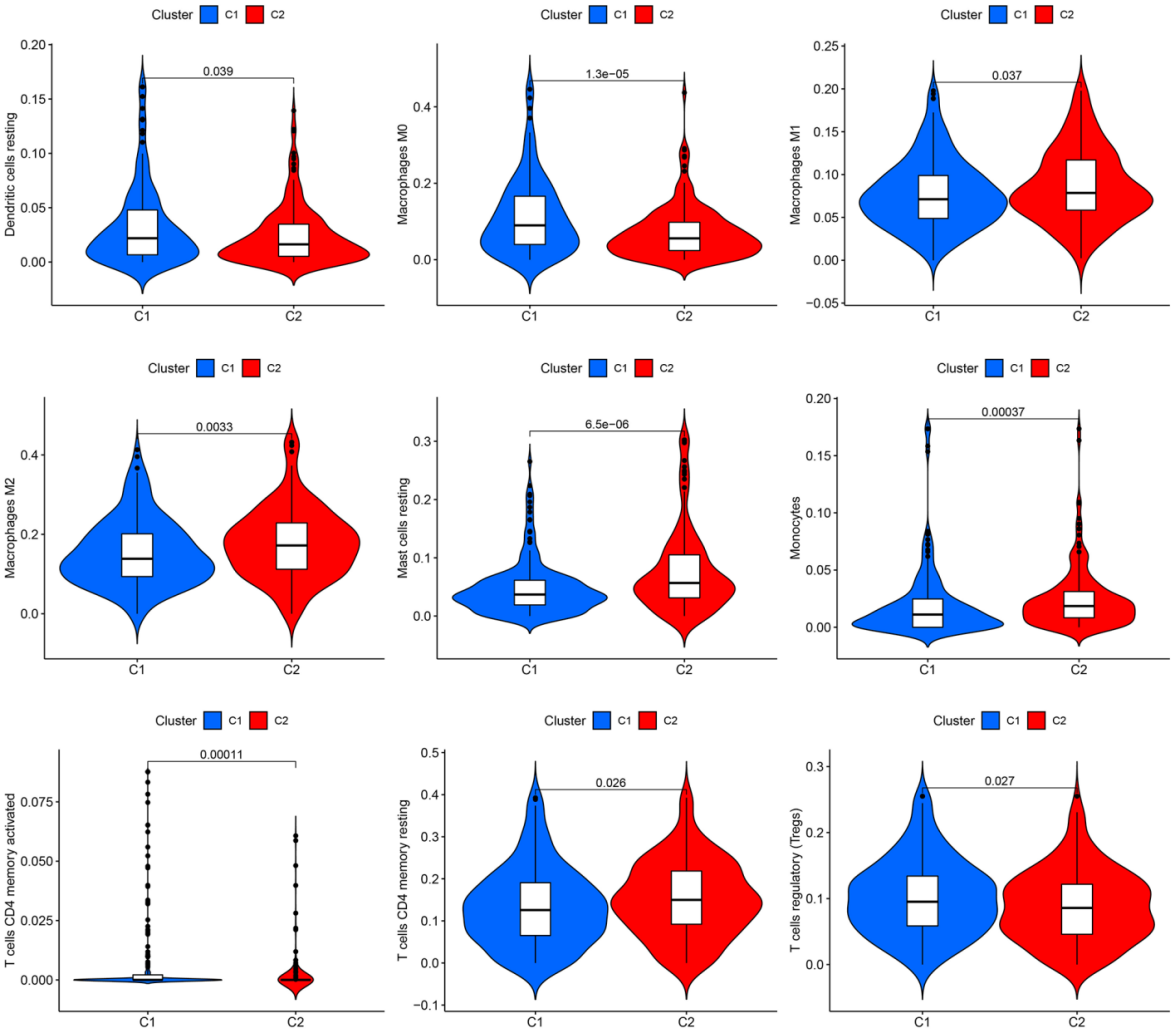
**Infiltration of immune cells in the two groups.** The two clusters display a violin plot illustrating dendritic cells, M0 macrophages, M1 macrophages, M2 macrophages, resting mast cells, monocytes, CD4 memory-activated T cells, CD4 memory resting T cells, and regulatory T cells (Tregs). Cluster 1 is referred to as C1, while Cluster 2 is known as C2.

According to the CIBERSORT algorithm, cluster 2 exhibited a notable increase in immune cell infiltration, specifically M1 macrophages, masting cells, M2 macrophages, Monocytes, and resting CD4 memory T cells, in comparison to cluster 1 (Figure 2).

### Identification and validation of a prognostic signature related to ubiquitination in HCC

**Figure 3:**
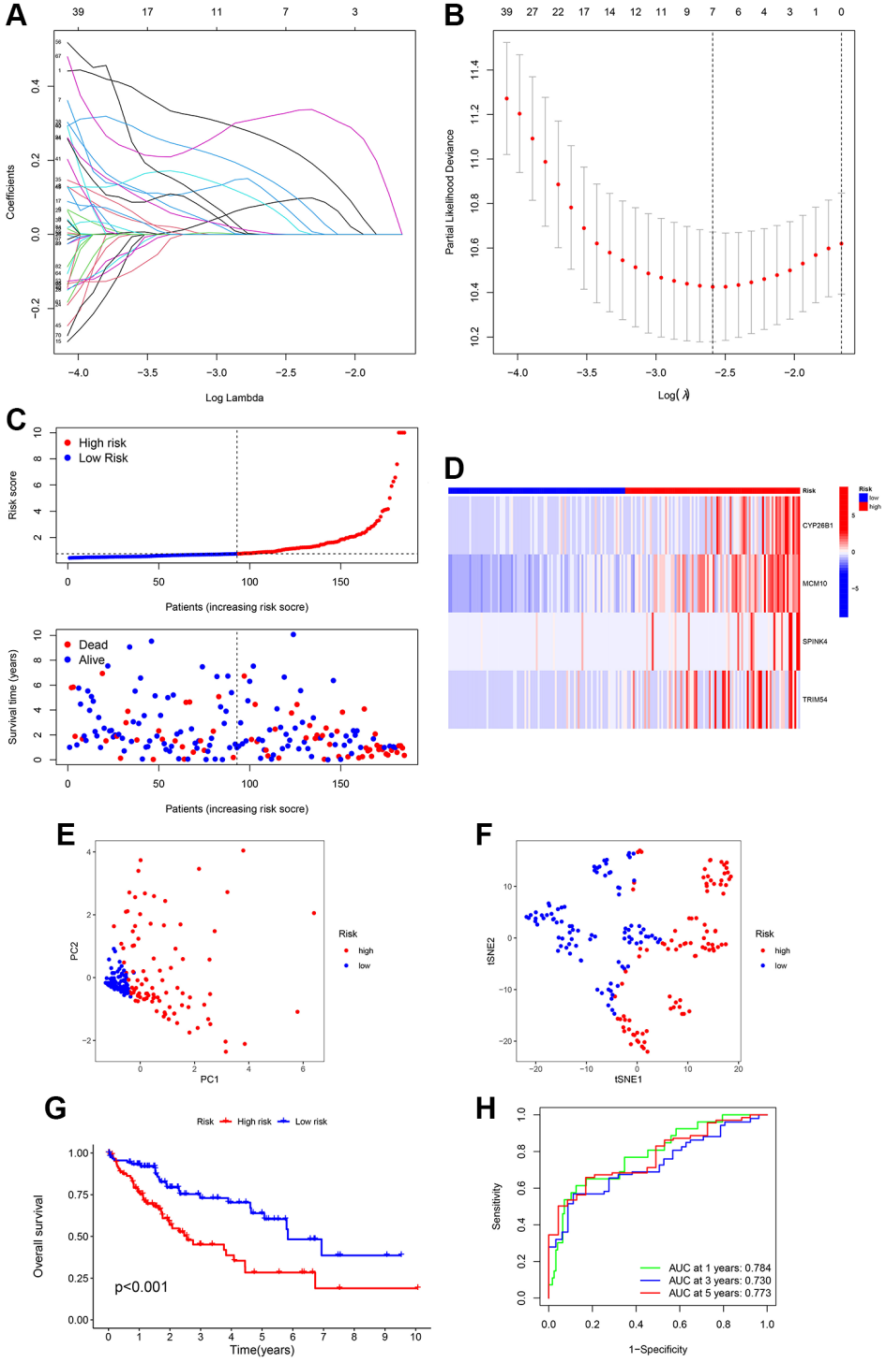
**Creation of a signature related to ubiquitination from four genes in various clusters of HCC patients.** LASSO Cox regression analysis penalizes genes that are expressed differentially (DEGs). (**A**, **B**) Cross-validation of potential genes using the lowest lambda value. (**C**) The risk score determines the survival time and status of every patient with HCC. (**D**) The expression of the four URGs in the low- and high-risk groups can be visualized through a heatmap. (**E**, **F**) Analysis of the signature using principal component analysis (PCA) and t-distributed stochastic neighbor embedding (t-SNE) was performed. (**G**) Comparative analysis of survival rates in two different risk subcategories. (**H**) The accuracy of the signature can be evaluated by analyzing the ROC curves for 1, 3, and 5 years.

Limma, a preset program of R, was utilized to identify ubiquitination-related DEGs to understand the biological function of the ubiquitination process. To understand the potential interrelation between survival rate and gene expression of patients, we analyzed the prognostic significance of DEGs during the progression of HCC. Univariate Cox regression analysis was utilized to examine the correlation between two survival clusters and gene expression levels. The analysis of LASSO Cox regression confirmed the strong gene signature associated with ubiquitination. Through LASSO analysis, a set of 7 genes was identified (Figure 3A, 3B) that can be utilized for the development of a prognostic model. Subsequently, a 4-gene signature (CYP26B1, MCM10, SPINK4, and TRIM54) was obtained by conducting a multiCox analysis. Based on Figure 3C, HCC patients were categorized into low- and high-risk groups using the median value. According to our maps indicating survival and risk status, the groups classified as high-risk experienced a higher number of fatalities compared to the low-risk groups. Figure 3D shows a heatmap that was created to exhibit the levels of expression of different expressed URGs in both groups. Furthermore, a significant distinction was noted between the two groups via t-SNE and PCA examination (Figure 3E, 3F). According to Figure 3G, the curve of Kaplan–Meier showed a notable variation in OS between the low- and high-risk groups (*p* < 0.001). To assess the predictiveness of the HCC signature, we generated ROC curves. The analysis of the ROC curve showed a notable prognostic impact for HCC using the risk model, with AUC values of 0.784, 0.730, and 0.773 for 1-, 3-, and 5-year survival, respectively (Figure 3H), indicating that patients who had low risk experienced better prognosis compared to those with high risk.

### The construction and verification of the risk signature

**Figure 4:**
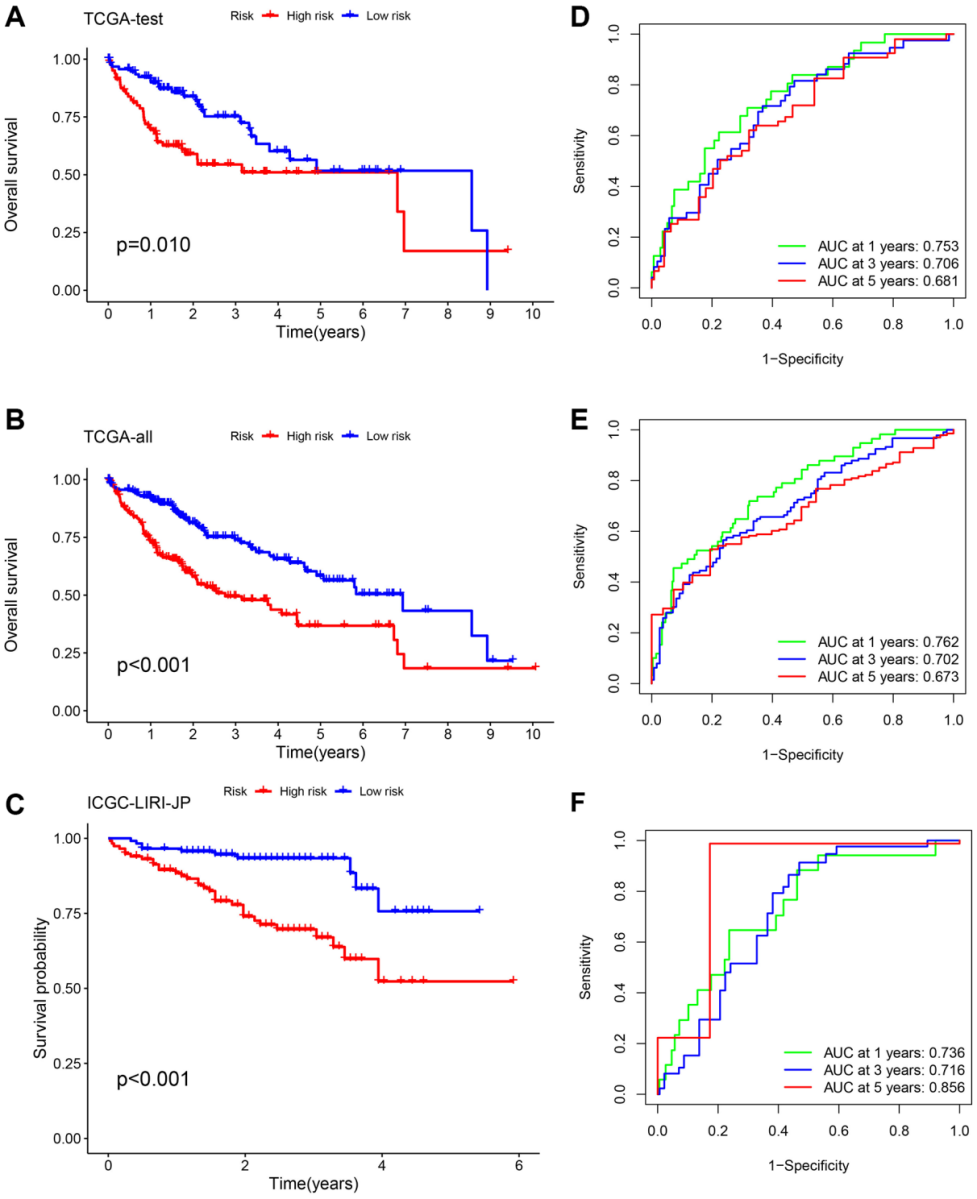
**Validation of the risk signature.** Survival analysis using the K-M method was performed on three different sets: the TCGA-test set (**A**, **D**), the TCGA-all set (**B**, **E**), and the ICGC-LIRI-JP set (**C**, **F**). To assess the precision of the signature, we utilized ROC curves for 1-, 3-, and 5-year intervals.

To ensure the stability and precision of the prediction signature, we evenly split all HCC patients in TCGA-LIHC into the internal training and test set. We obtained an additional microarray dataset (ICGC-LIRI-JP) from the ICGC database as an external test set for validating the risk-related signature. Survival analysis using the K-M method was performed on the internal-test set, internal-all set, and external-test set. We found that low-risk HCC patients had a considerably more favorable prognosis compared to high-risk HCC patients (*p* = 0.010, *p* < 0.001, and *p* < 0.001, respectively) (Figure 4A–4C). The analysis of the ROC curve demonstrated that the AUCs of survival rate in the internal-test set were 0.753 (1-year), 0.706 (3-year), and 0.681 (5-year). In the internal-all set, the AUCs were 0.762 (1-year), 0.702 (3-year), and 0.673 (5-year). Furthermore, in the external-test set, the AUCs were 0.736 (1-year), 0.716 (3-years), and 0.856 (5-years), indicating the consistent and reliable predictive ability of the signature (Figure 4D, 4E). These results further confirmed that the prognosis of high-risk patients was worse than low-risk patients.

### Evaluation of the practical usefulness of the prognostic indicator

**Figure 5:**
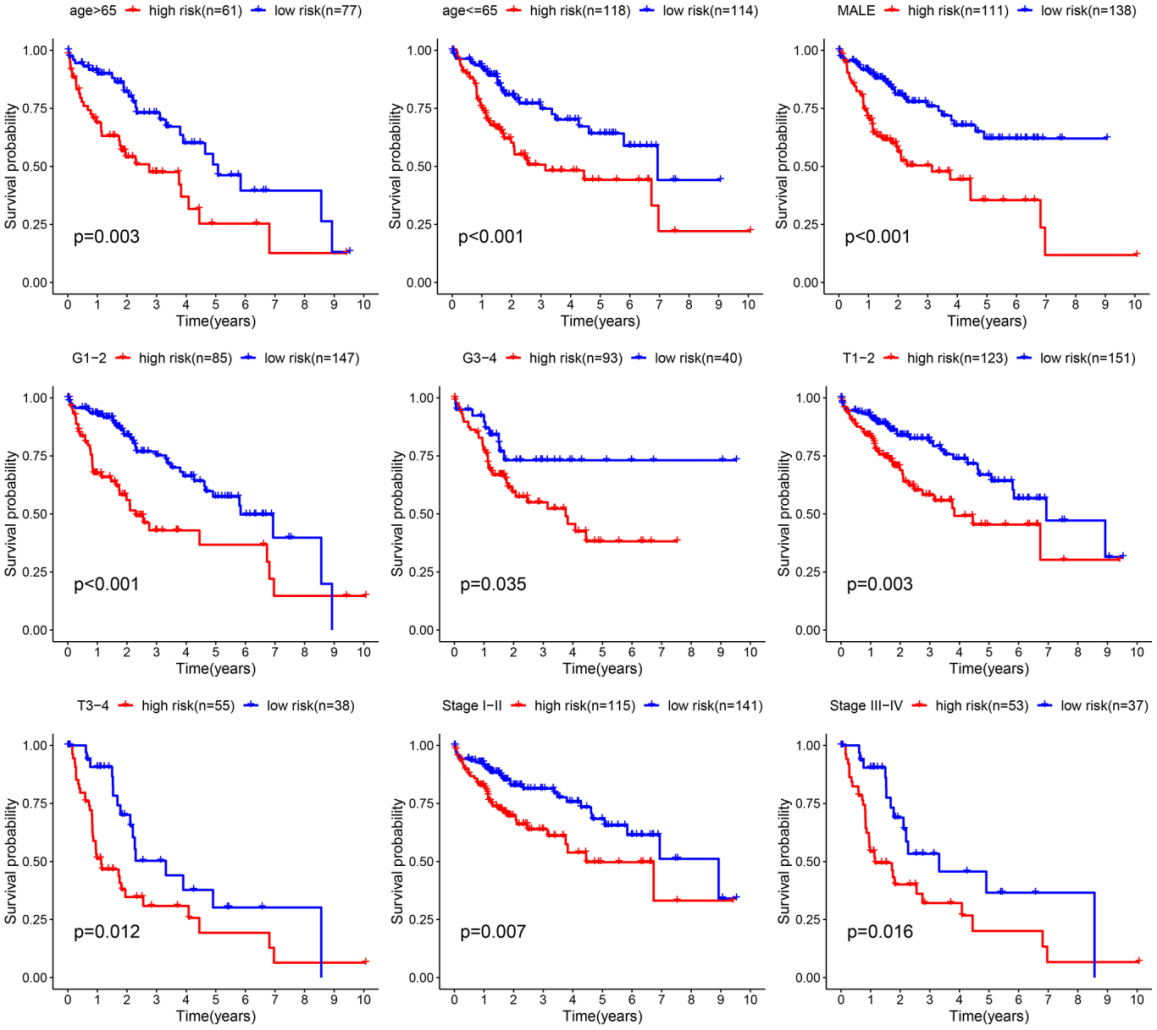
Comparative survival analysis among high- and low-risk groups across various clinical subgroups.

Afterward, an examination was conducted to explore the correlation between the clinical attributes and risk scores. Subgroup analysis was conducted to validate the trustworthiness of the risk model. We confirmed the variations in survival rates among different cancer subgroups, such as age, gender, Grade stage, T stage, and clinic stage (Figure 5). The results indicate that compared to the low-risk group, the high-risk group had a more unfavorable prognosis. Multivariate Cox analysis demonstrated that our risk-related signature possessed autonomous predictive significance. The existing analysis of additional stratification for different clinical features had also established robust prediction capabilities for signature.

### The construction and verification of the prognostic nomogram

**Figure 6:**
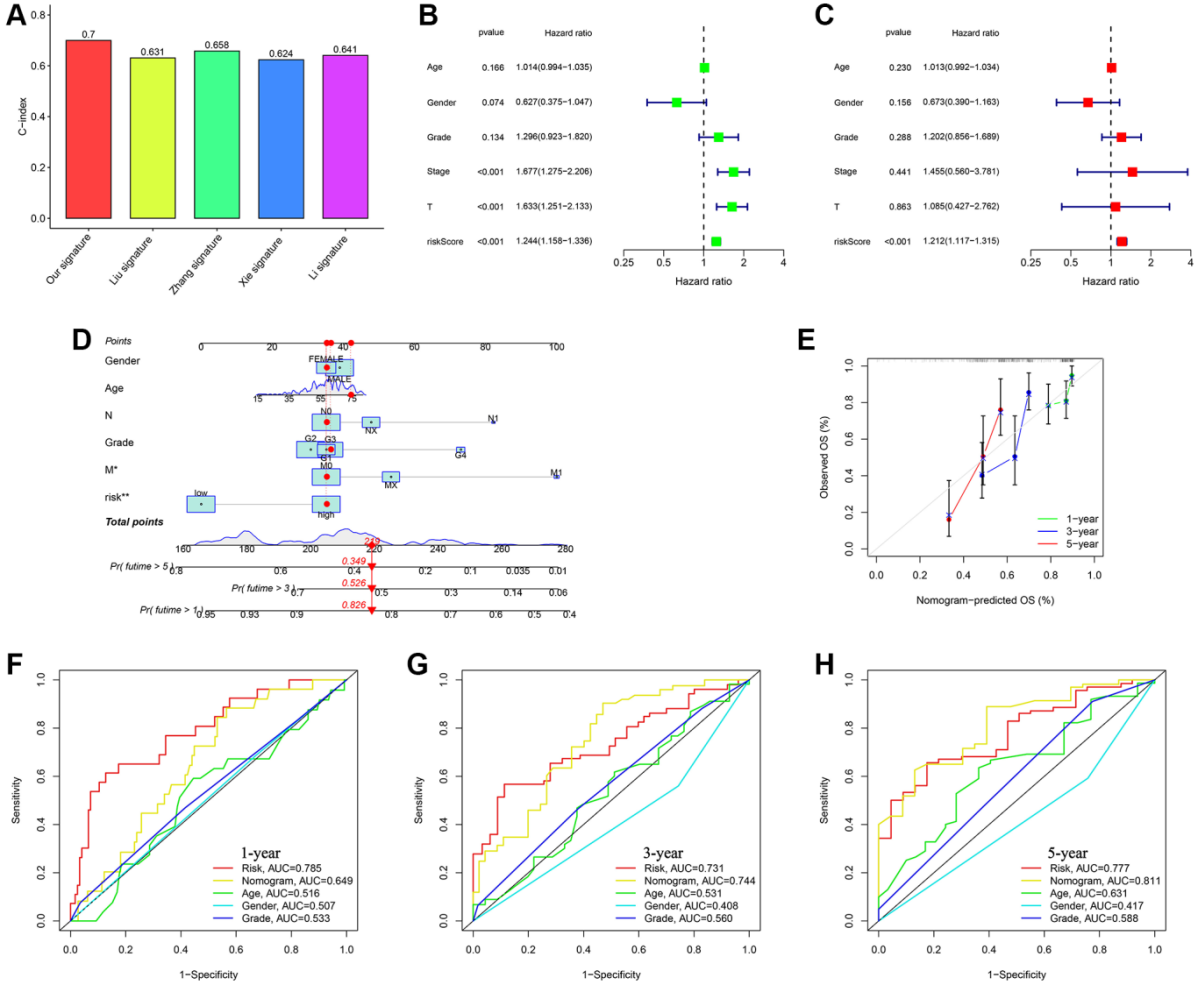
**Assessing the durability of the gene signature associated with ubiquitination and creating the nomogram.** (**A**) Evaluating four HCC models for comparison. (**B**) Cox analysis for TCGA-all set, considering only one variable at a time. (**C**) Cox analysis for TCGA-all set, considering multiple variables as independent factors. (**D**) The risk score and clinicopathological factors were used to create a predictive nomogram. (**E**) Calibration curves were created to compare the suggested nomogram with a perfect model. ROC curve analysis using multiple indices was conducted to examine the clinicopathological manifestations and nomogram for survival at 1-, 3-, and 5-year intervals (**F**–**H**).

Compared to another four published signatures for HCC, our signature had more advantages. To assess the precision and consistency of the predictive signature, we discovered that our signature had a concordance index (C-index) of 0.7, which outperformed the C-index values of the other four predictive signatures for HCC (0.631, 0.658, 0.624, 0.641, respectively) as shown in Figure 6A. Risk scores were considered as independent analytical indicators by using analyses of multivariate and univariate Cox regression (Figure 6B, 6C). A prognostic nomogram was utilized to generate distinct numerical probabilities for OS (Figure 6D), with considering various risk scores and clinical characters. We generated the calibration curve of the nomogram, and accurately predicted the 1, 3, and 5-year OS in comparison to the ideal model (Figure 6E). ROC curves were employed to assess the accuracy of the prognostic of the nomogram and the other signatures. As was shown in Figure 6F–6H, the AUC values for survival were 0.649 (1-year), 0.744 (3-year), and 0.811 (5-year). The nomogram had a stronger long-term predictive ability for prognosis than risk scores and other clinical characters.

### Analyzing the immune response variations within different subcategories

**Figure 7:**
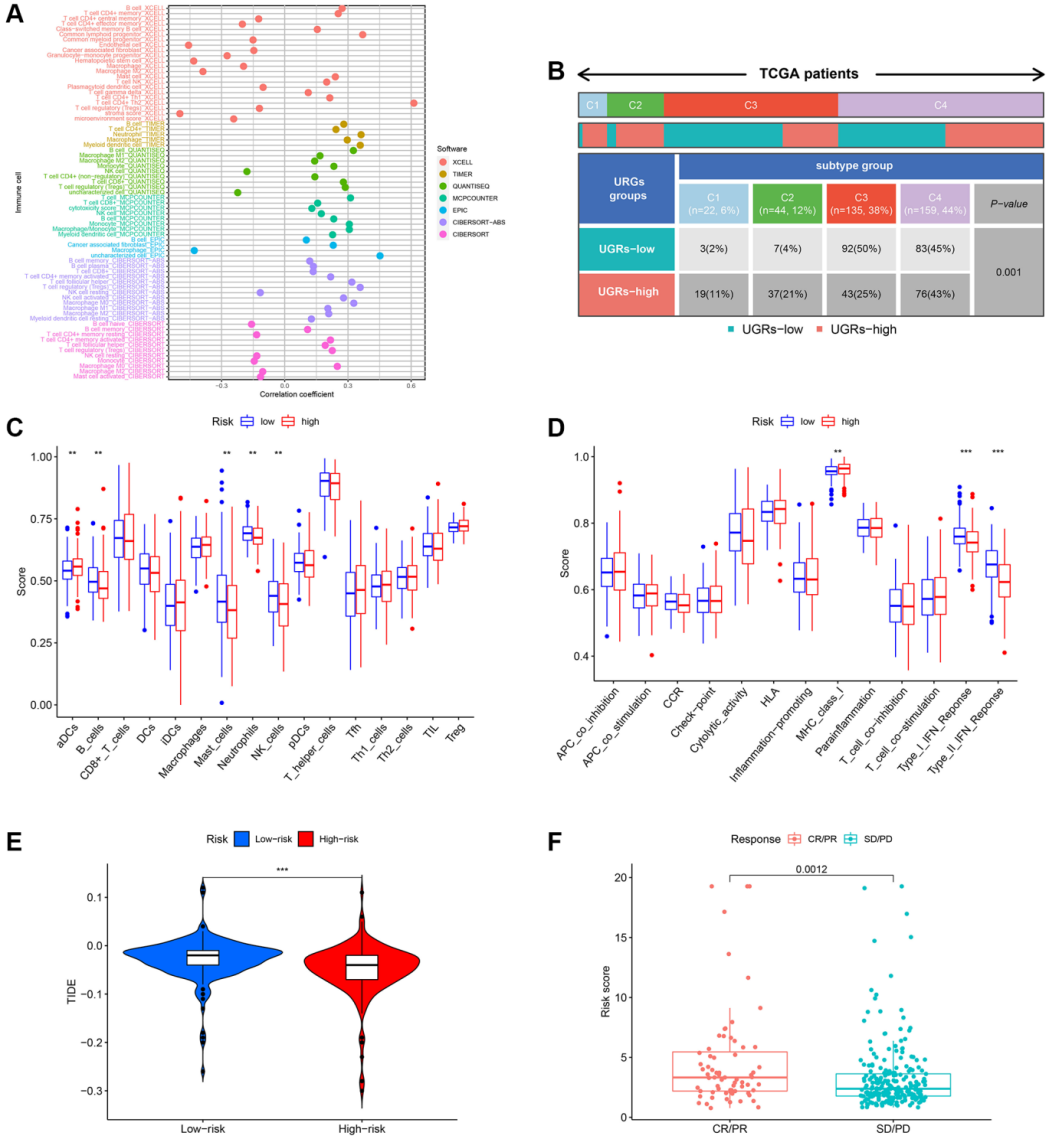
**Evaluation of the infiltration of immune cells.** (**A**) The XCELL, TIMER, QUANTISEQ, MCPCOUNTER, EPIC, CIBERSORT-ABS, and CIBERSORT algorithms were utilized to conduct correlation analysis between risk score and various immune cells. (**B**) The prevalence of TIME subtypes among TCGA patients. (**C**) Graphical representation of the infiltration of immune cells using a boxplot. (**D**) Boxplot illustrating the functionality of the immune system. (**E**) The TIDE score difference between the high-risk and low-risk groups. (**F**) Evaluating the risk score between the CR/PR and SD/PD groups. CR represents complete response, PR represents partial response, SD represents stable disease, and PD represents progressive disease.

Prior research has indicated that tumor microenvironment (TME) plays an important role in the progression of tumors. To examine the variations in immune-related annotations and immune cell infiltration among subtypes, we conducted ssGSEA. Using seven distinct algorithms, we estimated the variances in infiltration of immune cells among subtypes, as depicted in Figure 7A. The bubble chart demonstrated the correlation between the immune cells and risk score. According to the prior investigation, we categorized individuals with HCC into four subtypes of tumor immune microenvironment (TIME), which are immune activation (C1), immune suppression (C2), immune exclusion (C3), and immune residence (C4) phenotypes (as shown in Figure 7B). The C1 and C2 subtypes have a higher proportion of individuals in the group of high-risk, whereas the C3 and C4 subtypes have a higher proportion of individuals in the group of low-risk. In the low-risk subgroup, B cells, DC, CD8+ T cells, neutrophils, mast cells, NK cells, T helper cells, pDC, and TIL infiltrated more frequently, whereas aDCs, Th1 cells, iDC, Th2 cells, macrophages, Tfh, and Tregs infiltrated more frequently in the group of high-risk (Figure 7C). The group of low-risk typically exhibited greater significance in terms of CCR, cytolytic function, promotion of inflammation, response to Type I IFN, response to Type II IFN, and co-stimulation of T cells. Figure 7D demonstrates that the high-risk group typically exhibits greater importance in terms of other immune-related functions. High TIDE was also linked to the low-risk score (Figure 7E). Immunotherapy responses were significantly correlated with groups containing low- and high-risk samples, and it was observed that therapy responses were better in HCC patients classified as high-risk (Figure 7F).

### Conducting functional enrichment analysis for HCC with high and low risk

**Figure 8:**
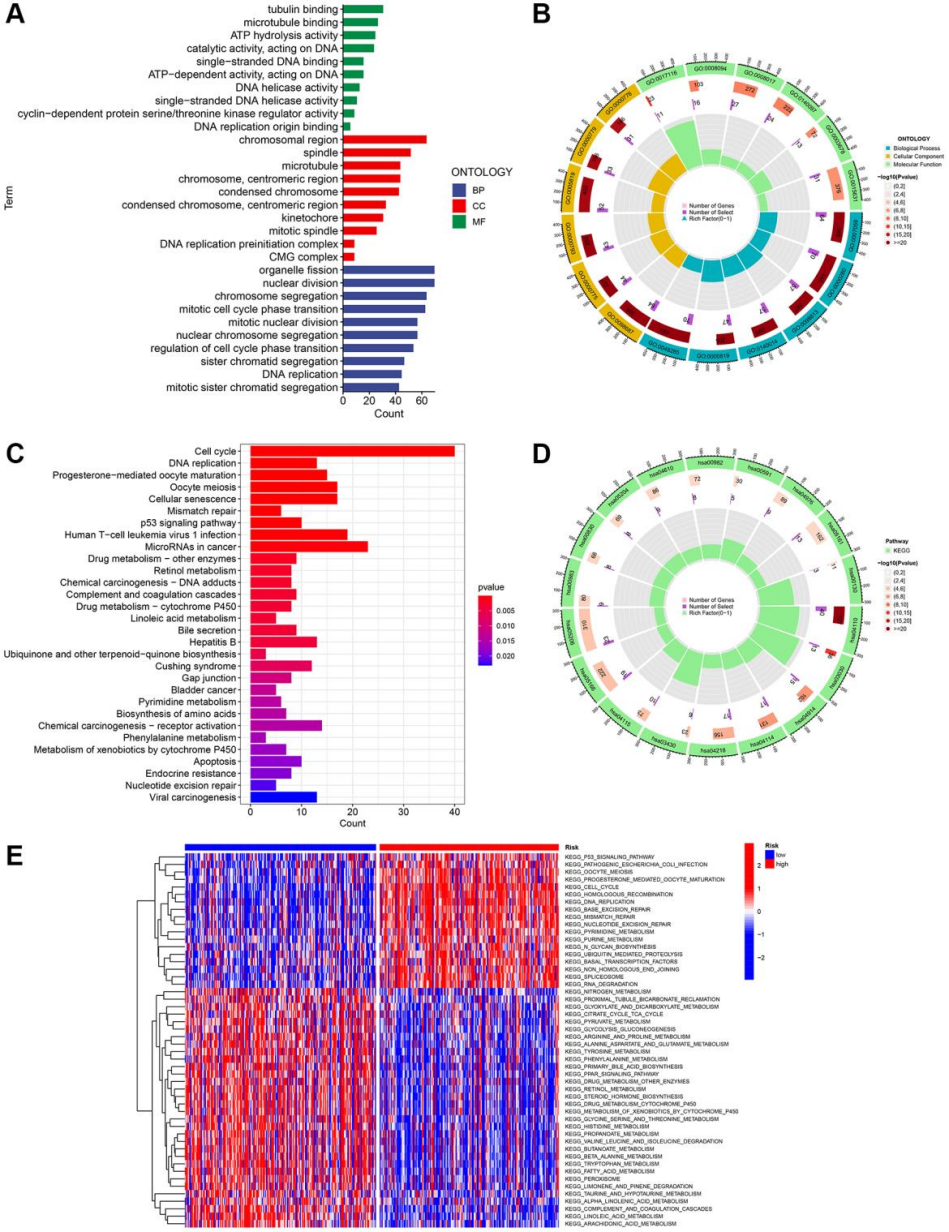
**Investigation of the gene signature related to ubiquitination in the entire TCGA dataset using functional analyses.** (**A**, **B**) Performing GO enrichment analysis on differentially expressed genes (DEGs) between the high-risk and low-risk groups. (**C**, **D**) Performing KEGG enrichment analysis on differentially expressed genes (DEGs) between the high-risk and low-risk groups. (**E**) Analysis of gene set variation (GSVA) in the high-risk and low-risk groups.

To further explore the presumed cellular role and pathway of HCC patients at high and low risk, the DEGs between the two subgroups were determined using the benchmarks of FDR < 0.05 and *p* < 0.05. According to the BP analysis, the three most enriched functions were processes related to the division of organelles, processes related to nuclear division, and processes related to chromosome segregation (Figure 8A). According to the CC analysis, the chromosomal region, spindle, and microtubule were identified as the three most enriched functions (Figure 8A, 8B). According to the MF analysis, it was confirmed that the functions of binding to tubulin, binding to microtubules, and hydrolyzing ATP were the most enriched activities (as shown in Figure 8A, 8B). Based on the KEGG analysis, the five most enriched pathways were cell cycle, microRNAs in cancer, cellular senescence, oocyte meiosis, and infection of human T-cell leukemia virus 1 (as shown in Figure 8C, 8D). These four URGs may utilize the P53 signaling pathway as a potential pathway. Furthermore, as depicted in Figure 8E, the GSVA heatmap revealed notable variations in enrichment functions between the high and low-risk cohorts.

### Anticipating responsiveness to chemotherapy medications

**Figure 9:**
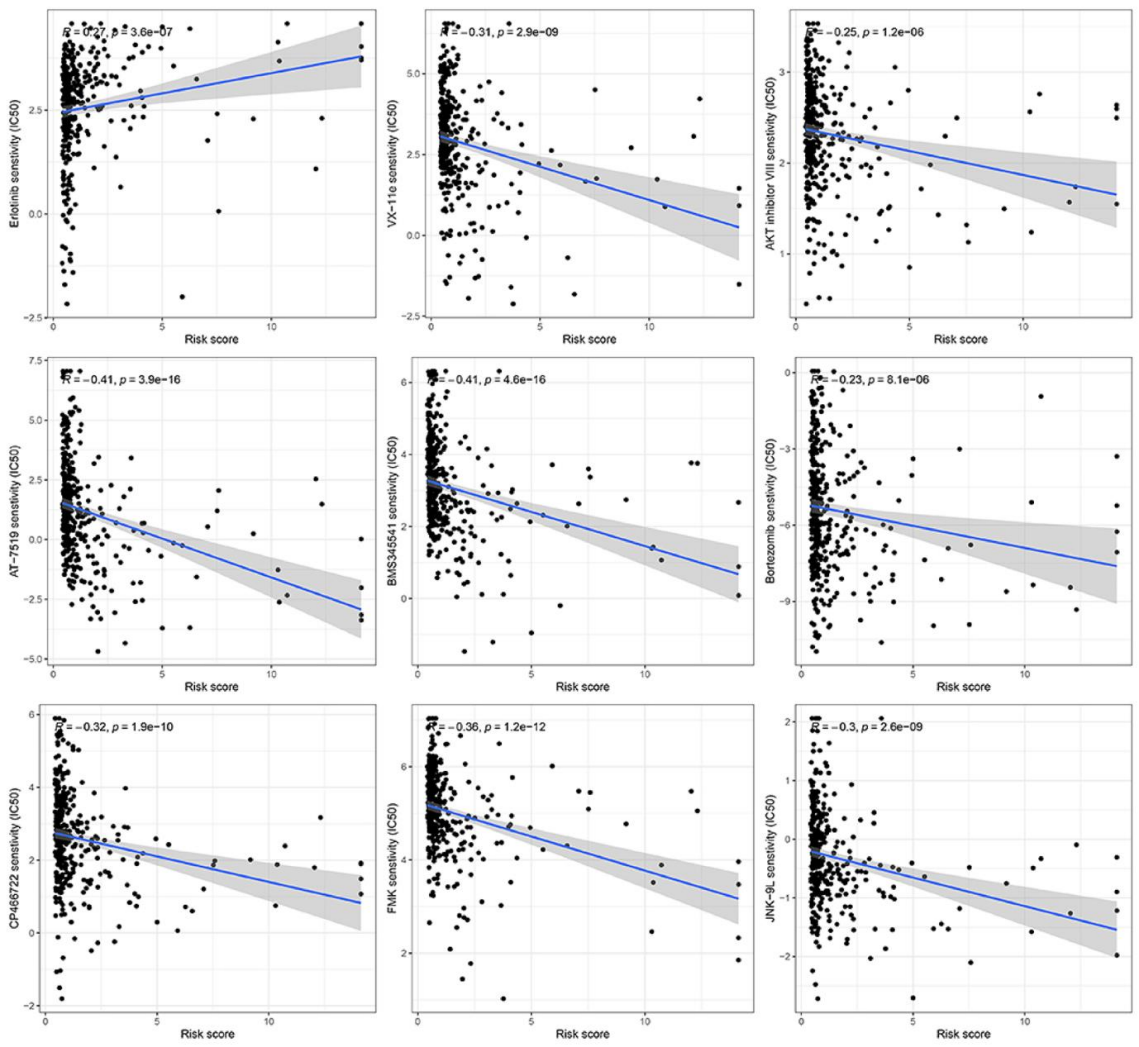
Prediction of the response to chemotherapy using a gene signature associated with ubiquitination.

At present, chemotherapy continues to be the primary approach for adjuvant treatment in patients diagnosed with HCC. Nevertheless, numerous individuals tend to acquire resistance to medications used in chemotherapy. In this present investigation, we anticipated the reaction of subsets to specific chemotherapy medications (Figure 9). The findings indicated that VX-11e, AKT inhibitor VIII, AT-7519, BMS345541, Bortezomib, CP466722, FMK, and JNK-9L exhibited greater effectiveness in treating low-risk HCC patients, implying potential benefits for this specific group of patients. Furthermore, it was discovered that HCC patients at high risk exhibited elevated estimated IC50s for erlotinib compared to those at low risk.

### Real-time quantitative polymerase chain reaction

**Figure 10:**
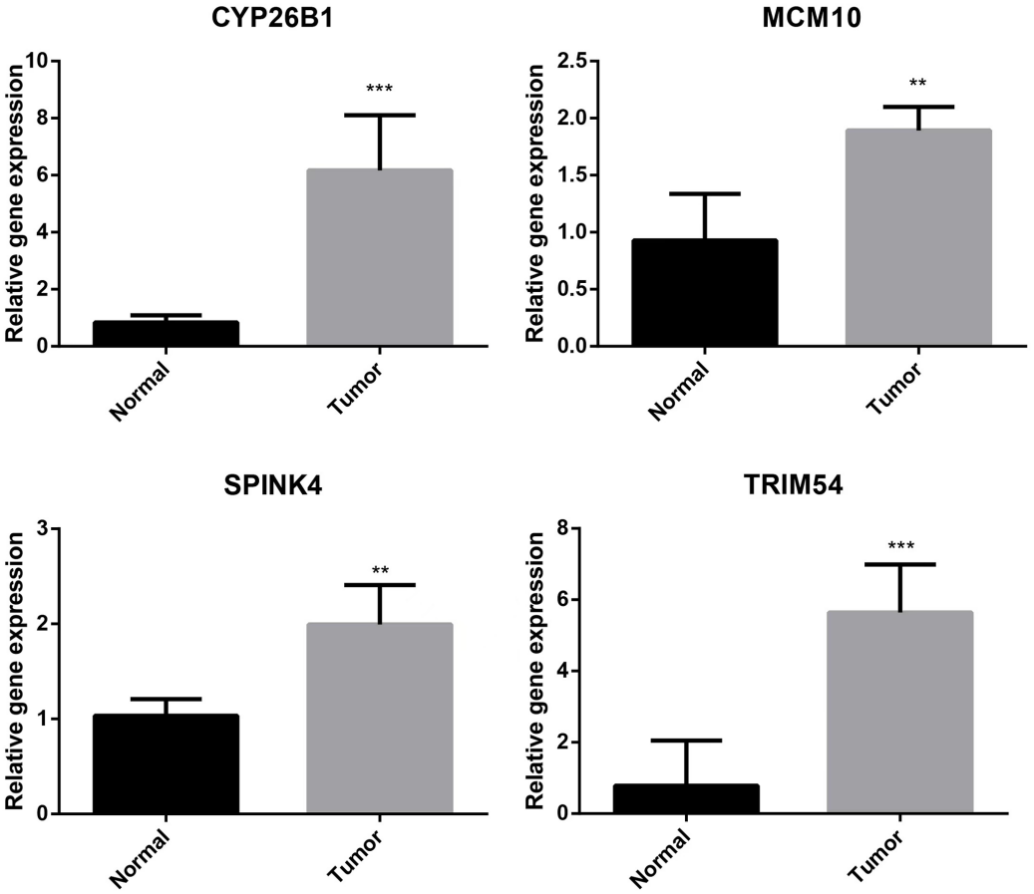
Validation of CYP26B1, MCM10, SPINK4, and TRIM54 through prognostic analysis and RT-qPCR.

Predictive biomarkers for HCC were confirmed to be four genes that formed the ubiquitination-related signature. As shown in Figure 10, the mRNA expression of CYP26B1, MCM10, SPINK4, and TRIM54 was compared and measured using RT-qPCR to determine transcription levels. Tumor tissues exhibited elevated expression levels of CYP26B1, MCM10, SPINK4, and TRIM54 compared to normal liver tissues, as indicated by the results.

### Immunohistochemistry of the UEGs in liver tissue

**Figure 11:**
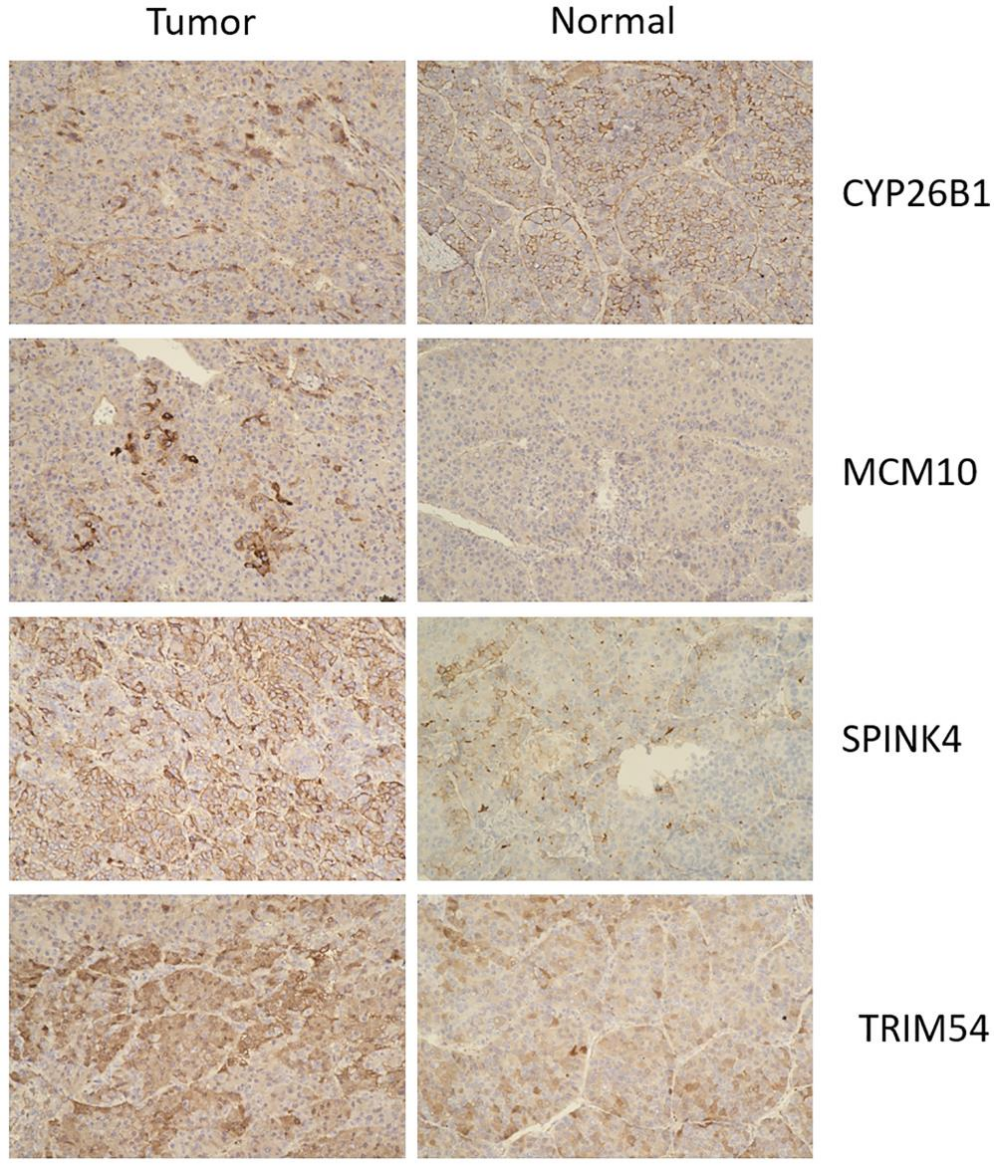
Immunohistochemistry of CYP26B1, MCM10, SPINK4, and TRIM54 expression in tumor tissue and normal tissue.

Immunohistochemistry analysis showed that CYP26B1, MCM10, SPINK4, and TRIM54 were highly expressed in tumor tissue compared to normal tissue (Figure 11). Tumor tissues exhibited high expression levels of CYP26B1, MCM10, SPINK4, and TRIM54 in the current study.

## DISCUSSION

HCC ranked among the most prevalent cancers globally, leading to significant mortality rates. It was highly significant to identify dependable and efficient biomarkers for the prognosis of HCC. Extensive research has been conducted on various genes expressed in HCC tissues. The growth and spread of tumors can be facilitated by cancer cells through the targeted inhibition or enhancement of certain mRNA translations, ultimately resulting in reduced survival rates for individuals with cancer. In a comprehensive HCC cohort, consisting of a validation dataset and a testing dataset, we discovered a unique signature associated with ubiquitination. This signature exhibited high levels of sensitivity and specificity.

The process of protein dynamic modulation associated with proliferation, cell growth, and survival was important for different cellular processes through ubiquitination. Prior studies have shown that E3 ubiquitin ligases and deubiquitinases play an important role in regulating tumor spread [42, 43]. Nevertheless, the precise function of protein ubiquitination in the HCC microenvironment remained unclear. Therefore, further investigation on URG in the TME is quite important for research on novel clinical treatment methods. In our current investigation, it was observed that HCC exhibited significant upregulation of CYP26B1, MCM10, SPINK4, and TRIM54, which were associated with an unfavorable prognosis. A novel prognosis prediction method of HCC was generated by constructing a 4-gene signature using DEGs from ubiquitination-related tumor classification. Additionally, a multifactorial analysis was employed to assess the signature’s significance in risk stratification, immune activity, and chemotherapy response among HCC patients.

For hepatocellular carcinoma (HCC), we performed a tumor classification using a 4-gene signature (CYP26B1, MCM10, SPINK4, and TRIM54) derived from differentially expressed genes (DEGs) associated with ubiquitination. The functions of these genes were significant in different types of cancers, including HCC. According to Wang and colleagues was discovered that CYP26B1 had a significant presence in the retinoic acid metabolic process and the retinol metabolism pathway, exerting a crucial influence on the advancement of cervical cancer across various age brackets [44]. According to Chen and colleagues was documented that oral squamous cell carcinoma exhibited a notable upregulation of CYP26B1 [45]. The overabundance of MCM10 was linked to unfavorable outcomes in cases of ovarian cancer and prostate cancer [46, 47]. The authors Hu et al. SPINK4 was discovered to enhance the growth of colorectal cancer cells while suppressing ferroptosis [48, 49]. TRIM54 was highly expressed in gastric cancer cells and tissues, and high expression of TRIM54 was correlated to decreased survival rate of patients with gastric cancer [50]. Despite the unknown connection between these genes and HCC, we showcased their ability to predict the prognosis of HCC and verified the unfavorable result of high-risk HCC determined by the signature derived from gene expression.

Although multiple staging and prognostic systems have been developed, none are universally applicable or agreed upon for predicting survival, and most of them do not include effective biomarkers. As was previously published, Liu et al. [20] found that mitophagy was closely related to the immune microenvironment, immune checkpoint (IC) related genes, Cancer stem cells (CSCs), and prognosis in HCC patients. They constructed a signature containing four mitophagy-related genes (MRGs) to predict the prognosis of HCC patients. However, the results have not been further elucidated and validated *in vitro* and *in vivo*. Zhang et al. [21] constructed and validated a stemness-hypoxia-related prognostic signature that can be used to predict the efficacy of IC inhibitor therapy. They also verified that C7, CLEC1B, and CXCL6 were indeed associated with stemness and hypoxia through a hypoxic cell model. Similarly, there was no result of *in vitro* and *in vivo* experiments. Xie et al. [22] constructed and validated a prognostic risk model based on 5 cuproptosis-related immune checkpoint genes (CRICGs). In the meantime, they evaluated the potential correlation between the signature and clinicopathological characteristics, tumor immunity, and somatic mutation. In this paper, they applied the cell lines for expression level validation. Li et al. [23] established a new prediction signature of eight genes related to cuproptosis and the tricarboxylic acid cycle (TCA) process. These research findings had their advantages and disadvantages, but our signature had a higher C-index of 0.7, which outperformed the C-index values of the other four predictive signatures for HCC (0.631, 0.658, 0.624, 0.641, respectively).

Two clusters of ubiquitination were created and validated in our research to enhance the prediction of HCC outcomes. Cluster 2 had a better prognosis compared to Cluster 1. Cluster 1 exhibited higher clinic grade, pathological stage, and T stages compared to cluster 2, suggesting a potential correlation between cluster 1 and an elevated tumor stage of HCC.

Upon comparing the immune cell infiltration in the two clusters, it was observed that cluster 2 exhibited significant enrichment of M1 macrophages, M2 macrophages, resting CD4 memory T cells, masting cells, and Monocytes. The analysis of LASSO Cox regression confirmed the strong gene signature associated with ubiquitination. The patients who had low risk experienced better prognosis compared to those with high risk. The positive performance of the signature was confirmed by the ROC curves at 1, 3, and 5-year intervals. To ensure the stability and precision of the prediction signature, we evenly split all HCC patients in TCGA-LIHC into the internal training and test set. To validate the risk-related signature, we obtained an additional microarray dataset (ICGC-LIRI-JP) from the ICGC database as an external test set. The existence analysis of additional stratification for different medical subgroups has also established robust prediction capabilities for features. Both multivariate and univariate Cox analysis demonstrated that our risk-related signature possessed autonomous predictive significance. Compared to another four published signatures for HCC, our signature had more advantages. Next, we utilized a nomogram approach to enhance the effectiveness of the prognostic indicator by taking into account clinical characteristics such as age, gender, clinical grade, pathological stage, T stages, and risk scores. Overall survival of HCC patients was accurately predicted by this significant model.

Afterward, we conducted a functional examination of DEGs between the low and high-risk groups to investigate the potential pathways and functions associated with the 4-gene signature. The GO enrichment analysis showed that these DEGs were associated with the ‘chromosomal region’, ‘organelle fission’, and ‘nuclear division’, indicating a correlation between these DEGs and cell proliferation. The outcome aligned with the KEGG enrichment analysis, indicating notable enrichment of terms such as ‘cell cycle’, ‘microRNA in cancer’, and ‘P53 signaling pathway’. Tumorigenesis is primarily driven by abnormalities in the progression of the cell cycle. An increasing amount of evidence suggests that the cell cycle regulatory pathway connects with other characteristics of cancer, such as alterations in metabolism and evasion of the immune system. Therefore, these four differentially expressed genes (DEGs) could potentially serve as viable targets for anti-cancer treatment.

The role of TME is crucial in HCC immunotherapy. Examining TME can enhance our comprehension of how ubiquitination impacts the prognosis of individuals with HCC. Consequently, we evaluated the distribution of different immune cells in HCC by employing six widely utilized algorithms. B cells, DC, CD8+T cells, mast cells, NK cells, neutrophils, T helper cells, pDC, and TIL significantly infiltrated the TME in patients with low risk. Regulating the immune response against tumors, these immune cells have the potential to impact the development of HCC. Additional investigations uncovered that the low-risk group exhibited enrichment in CCR, cytolytic function, promotion of inflammation, co-stimulation of T cells, response to Type I IFN, and response to Type II IFN. Furthermore, we assessed the efficacy of specific chemotherapy treatments for various subcategories of HCC. The findings indicated that patients classified as low-risk exhibited greater estimated IC50 values for 8 chemotherapy medications (VX-11e, AKT inhibitor VIII, AT-7519, BMS345541, Bortezomib, CP466722, FMK, and JNK-9L) compared to those classified as high-risk. The potential of these findings can help in determining the choice of treatment for every patient with HCC.

The diversity of HCC can be observed through our distinctive mark in terms of ubiquitination, and we validate its potential as a predictive tool for survival rate, immune activity, and treatment response.

To summarize, our research discovered a four-URG pattern linked to immune infiltration and responsiveness to medication. The accuracy of the prediction model was verified by utilizing both training and test sets. The findings indicated that this unique marker has the potential to serve as a new indicator for predicting survival and prognosis, offering an individualized approach to treating HCC. Nevertheless, the precise mechanism of URG remains undisclosed and necessitates further investigation.

## Supplementary Materials

Supplementary Tables
