# Supplementary material for: Prognostic implication and immunotherapy response prediction of a novel ubiquitination-related gene signature in liver cancer
Source: Aging (Albany NY). 2024 Jun 12;16(11):10142–64. doi: 10.18632/aging.205926 (PMC11210240; doi:10.18632/aging.205926)
Supplement: Supplementary Tables [file aging-16-205926-s001.pdf]

SUPPLEMENTARY TABLES

Supplementary Table 1. Clinical information such as age, sex, clinic grade, pathological stage, and T stage, were obtained from the Cancer Genome Atlas (TCGA) LIHC project.

Covariates	Type	Total	Test	Train	*P*-value
Age	≤65	232 (62.7%)	105 (56.76%)	127 (68.65%)	0.024
Age	>65	138 (37.3%)	80 (43.24%)	58 (31.35%)	
Gender	FEMALE	121 (32.7%)	61 (32.97%)	60 (32.43%)	1
Gender	MALE	249 (67.3%)	124 (67.03%)	125 (67.57%)	
Grade	G1	55 (14.86%)	23 (12.43%)	32 (17.3%)	0.2791
Grade	G2	177 (47.84%)	97 (52.43%)	80 (43.24%)	
Grade	G3	121 (32.7%)	57 (30.81%)	64 (34.59%)	
Grade	G4	12 (3.24%)	5 (2.7%)	7 (3.78%)	
Grade	Unknow	5 (1.35%)	3 (1.62%)	2 (1.08%)	
Stage	Stage I	171 (46.22%)	85 (45.95%)	86 (46.49%)	0.5399
Stage	Stage II	85 (22.97%)	44 (23.78%)	41 (22.16%)	
Stage	Stage III	85 (22.97%)	45 (24.32%)	40 (21.62%)	
Stage	Stage IV	5 (1.35%)	1 (0.54%)	4 (2.16%)	
Stage	Unknow	24 (6.49%)	10 (5.41%)	14 (7.57%)	
T	T1	181 (48.92%)	89 (48.11%)	92 (49.73%)	0.8288
T	T2	93 (25.14%)	48 (25.95%)	45 (24.32%)	
T	T3	80 (21.62%)	41 (22.16%)	39 (21.08%)	
T	T4	13 (3.51%)	5 (2.7%)	8 (4.32%)	
T	Unknow	3 (0.81%)	2 (1.08%)	1 (0.54%)	
M	M0	266 (71.89%)	135 (72.97%)	131 (70.81%)	0.1313
M	M1	4 (1.08%)	0 (0%)	4 (2.16%)	
M	MX	100 (27.03%)	50 (27.03%)	50 (27.03%)	
N	N0	252 (68.11%)	121 (65.41%)	131 (70.81%)	0.5738
N	N1	4 (1.08%)	2 (1.08%)	2 (1.08%)	
N	NX	113 (30.54%)	61 (32.97%)	52 (28.11%)	
N	Unknow	1 (0.27%)	1 (0.54%)	0 (0%)	

Supplementary Table 2. A set of 79 genes related to ubiquitination (URGs) was obtained from the MSigDB database.

BCL10
CDC34
CDC73
CTR9
DERL1
H2BC1
H2BC10
H2BC11
H2BC12
H2BC13
H2BC14
H2BC15
H2BC17
H2BC3
H2BC4
H2BC5
H2BC6
H2BC7
H2BC8
H2BC9
HLA-A
HLTF
LEO1
OTULIN
PAF1
PCNA
PEX10
PEX12
PEX13
PEX14
PEX2
PEX5
PRKDC
RAD18
RNF144A
RNF152
RNF181
RNF20
RNF40
RPS27A
RRAGA
RTF1
SELENOS
SHPRH
TMEM129

UBA1
UBA52
UBA6
UBB
UBC
UBE2A
UBE2B
UBE2C
UBE2D1
UBE2D2
UBE2D3
UBE2E1
UBE2E3
UBE2G1
UBE2G2
UBE2H
UBE2J2
UBE2K
UBE2L3
UBE2N
UBE2Q2
UBE2R2
UBE2S
UBE2T
UBE2V2
UBE2W
UBE2Z
UCHL3
USP5
USP7
USP9X
VCP
WAC
WDR61
